# Gleanings

**Published:** 1893-09

**Authors:** F. H. Lutze

**Affiliations:** Brooklyn, N. Y.


### GLEANINGS.

F. H. Lutze, M. D., Brooklyn, N. Y.

### Eyes.

As if falling from sockets. Tril.

— — — pulled out. Natr-c.

— — — starting from sockets. Bell.

— pain in, < lying on left side. Lac-fel.

— — — < — on painful side. Syphilin.

— — — > — — — —. Zinc.

— — — right eyeball, extends to right forehead and temple < p. m. Badiaga.

Visions on closing eyes. Apis, Argent-n., Ars., Atrop., Bell., Bry., Calc-c., Camph., Caust., Chloral, China, Coccul., Euphras., Gels., Graph., Helleb., Ignat., Laches., Led., Lycopod., Natr-m., Natr-ars., Petrol., Plumb., Puls., Sambuc., Seraph., Sepia, Spong., Stram., Sulph., Tarent., Thuja.

Tears, absence of in children four months old usually denotes a fatal disease.

On opening eyes terrified to spasms. Stram.

Like needles through left eyeball. Spigel.

Quivering of left upper lid, especially in convulsions of children. Arum-tri.

Vision obscured as by smoke. Gels., Crocus.

A straw appears to him so large he lifts his foot up high in order to step over it. Agaricus.

Lashes of eyes turn inward toward eye and inflame it, especially at outer canthus, where margins are very sore. Borax, Silicea, Puls.

Eyes seem to turn in head ; roll up all the time ; pain in right eye and right forehead. Gels.

— fixed on dark sides of the room away from the light. Stramon.

— smarting at external canthi, which are red and prick at times. Sulph.

Yellow or greenish-yellow halo around the light. Sulph.

Injuries to eyes. Acon., Symphyt.

### Ears.

Earache in babies; they want to be carried slowly; cry sad and heartbroken ; $<$ heat, $>$ cold air. Puls.

— ache. Child cries lustily $>$ heat, and pressing ear against nurse's breast. Cham.

Distention of ears. Bell., Kali-iod., Laurocer., Nitr-ac.

Otitis with fish-brine odor. Tellur.

— — putrid meat odor. Thuja.

Hearing acute only for human voice. Ignat.

— dull for human voice. Phos.

Deafness from concussion or sustained shock as from explosion of cannon. Arnic.$^{30}$, Strychnin.$^{6}$.

— from fatigue. Picr-acid.

— — worry or mental shock or hard work. Magn-c.

— to distant sounds. Tannic-ac.

— due to a culmination of all above. Chinin-sulph.$^{30}$ not lower potency.

Hearing water run from a hydrant turns him sick; must leave table. Hydrophob.

Inflammation, acute, of middle ear. Acon., Bell., Cham., Fer-phos., Gels., Puls.

— chronic, catarrhal of middle ear. Baryt-m., Iod., Kali-mur., Kali-sulph., Merc-dul., Merc-iod-rub.

— of mastoid process. Caps., Hep., Sil.

Chronic otitis media suppurativa. Calc-c., Calc-phos., Calc-sulph., Elaps, Kali-sulph., Merc., Silicea, Sulph., Teucrium.

Neuralgia of ear. Cham., Dulc., Plantag-maj.

— — — from getting wet. Silicea.

— — — — carious teeth. Plant-m.

Congestion, cerebral, from diseases of middle ear. Bell., Cicuta-vir., Coccul., Fer-phos., Gels., Glon., Op., Veratr-vir.

Buzzing and roaring in ears. Chin-s.

— — — — with dizziness. Digit.

Roaring in ears, with difficult hearing. Soda-salicyl.

Re-echo, sounds re-echo in ears. Caust., Kali-brom., Nitric-ac., Nux-v., Glon., Phos., Phos-ac., Sarsapar., Therid., Secale.

Roaring, ringing, hissing, or singing in ears or tinkling; Chin-sulph.

Ringing and tinkling with deafness as if the ear was stopped up by a membrane. Carbon-s., Graph., Hydrastis-c.

Sensitiveness to 32-feet organ pipes. Chenopod-anthelmint.

Hissing in ears, > by inserting finger and drawing parts asunder. Æthus.

Sensation of wind rushing out of ears. Chenopodium-glauci.

— — cold wind blowing into the right ear. Causticum, Mang., Meny., Plat., Staph.

Hearing better, when riding in the car or a carriage. Graph., Puls.

Hammering and tearing in ears, *evening in bed till after* midnight, with micturition every half-hour, and coldness of legs up to knees. Thuja.

Otalgia, discharge ichorous, scalding pain pressing as from a plug. Silicea.

### Nose.

Fan-like motion of alæ-nasi. Ant-tart., Bapt., Brom., Ammoniacum., Chelidon., Lycopod., Phos.

Lump in posterior nose, sensation of. Wyethia.

Odor before nose as of an old catarrh, only subjective (to patient). Sulph.

— — — abominable to every one about. Merc.

Pressure on root of nose. Kali-bich., Nux-vom., Sticta-pulm.

Eruption, herpetic on end of nose. Æthusa.

Snuffles of children. Amm-c., Lycopod.

Cannot breathe through nose, must breathe through mouth. Amm-c.

Picks nose constantly, which bleeds easy, picks finger tips, does not like to answer questions. Conium.

Stuffed feeling at root of nose. Kali-bich., Nux-v., Sticta-p.

Nose sensitive, when inhaling cold air. Psor. (Actea, Cistus.)

Sensation of a lump in posterior nares, with dryness. Wyethia.

On sneezing or blowing nose, a pain in a hollow tooth. Thuja.

### Face.

Chapped lips. Alum., Arn., Chin., Graph., Nux-mosch.

Cracks in middle of upper lip. Bell.

— — — — lower lip. Cham., Hep., Puls., Phos.

Flushes of heat. Cimicif., Sulph., Sang., Veratr-vir., Ambr-gris., Oleum-an., Puls.

Hydroa on lips, at first clear, tending to yellow amber color, in clusters. Rhus-t.

Hydroa on lips, whiter, more pearly and singly. Natrum-mur.

Lips, peeling off. Thuja, Sulph-ac.

— — — painful. Nux-vom.

— picking, and nose, etc. Arum-tri., Con., Hell., Lachesis, Selen., Stront., Bell.

— scaling off. Berberis.

Tickling on face as of a hair. Laurocer.

Face pale, hippocratic during uterine hæmorrhage. China.

Ulcers or large sores on right side of nose. Ranunculus-scel.

Face wrinkled, with violent speech. Stram.

Erysipelas of left side of face or whole face, pain < laughing. Borax.

Flushes of heat, followed by profuse, warm perspiration. Sulph.

— — — — — — — — which soon becomes cold. Lachesis.

— — — associated with mental depression, as if a cloud had settled over the patient; distressed and suspicious without cause. Cimicif.

Yellow color about the mouth. Agar., Ars., Cina, Magnes-m., Sepia.

Erysipelas from right to left side of face. Apis.

— — left to right. Rhus-t.

— with delirium. Stram.

